# Exploring the Stability and Substrate Profile of Transaminase from *Silicibacter pomeroyi* with Ancestral Sequence Reconstruction

**DOI:** 10.1002/cbic.202500155

**Published:** 2025-05-30

**Authors:** Luyao Zhao, Bhu‐Bhud Thongrakon, Trishnamoni Gautom, Viktor Sahlberg, Per Berglund

**Affiliations:** ^1^ Department of Industrial Biotechnology School of Engineering Sciences in Chemistry, Biotechnology and Health KTH Royal Institute of Technology SE‐10691 Stockholm Sweden

**Keywords:** ancestral sequence reconstruction, *Silicibacter pomeroyi*, stability, ω‐transaminase

## Abstract

Amine transaminases (ATAs), belonging to the class III transaminases within the superfamily of pyridoxal‐5′‐phosphate‐dependent enzymes, catalyze transamination reactions between amino donors and amino acceptors. These enzymes are particularly appealing for their role in stereospecific synthesis of chiral amines. However, the stability of most ATAs is not satisfying, limiting their suitability for industrial applications. Among them, the amine transaminase from *Silicibacter pomeroyi* (*Sp*‐ATA) has drawn attention due to its high activity and broad substrate scope under mild conditions and high pH. Nevertheless, maintaining the activity at higher temperatures is a challenge. Previous studies to enhance enzyme function through directed evolution have shown promising results, yet predicting the cooperative effects of individual stabilizing mutations remains challenging. An alternative strategy is ancestral sequence reconstruction (ASR), which is based on gene sequences to create a more or less artificial phylogenetic tree. This study aims to leverage ASR techniques to explore the thermostability, solvent tolerance, and substrate profile of *Sp*‐ATA, to find more stable transaminases. By using *Sp*‐ATA as a template and incorporating insights from ancestral sequences, this strategy offers a promising approach for developing robust biocatalysts suitable for industrial applications.

## Graphical abstract

This study describes the successful identification of ancestral sequences N50 and N49, which have a longer half‐life of approximately four and two times the wild‐type *Sp*‐amine transaminase, respectively.. This approach effectively enhances the thermal stability of the transaminase.

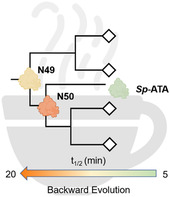

## 1 Introduction

Biocatalysis has emerged as an important technology in recent years, especially in asymmetric synthesis, due to the stereoselectivity, regioselectivity, and chemoselectivity of the biocatalysts, which make the process more environmentally friendly and simplified.^[^ ^1^, ^2^, ^3^, ^4^, ^5^, ^6^ ^]^ Chiral amines and their derivatives are widely found in many active pharmaceutical ingredients (API) and fine chemicals.^[^ ^7^, ^8^, ^9^ ^]^ They are high‐value synthetic targets, as these compounds often contain one or more stereocenters, and controlling the overall stereochemistry is quite challenging. Biocatalysis provides a more sustainable route for the synthesis of high‐purity chiral amines and their derivatives.^[^ ^3^, ^10^, ^11^ ^]^ Currently, biocatalysts used for the production of chiral amines mainly include hydrolases,^[^ ^12^, ^13^ ^]^ oxidoreductases,^[^ ^14^, ^15^ ^]^ and transferases.^[^ ^16^ ^]^ Amine dehydrogenases (AmDHs) and imine reductases (IREDs) have emerged with significant research focus over the past decade due to their catalytic role in the amination of ketones.^[^ ^17^, ^18^, ^19^, ^20^ ^]^ Despite their potential as alternatives to traditional methods for chiral amine synthesis, transaminases still hold significant potential for further investigation.^[^ ^21^, ^22^, ^23^ ^]^ Transaminases (TAs, EC 2.6.1.X) are enzymes that use the coenzyme pyridoxal 5′‐phosphate (PLP, a derivative of vitamin B6) to catalyze the transfer of amino groups to carbonyl groups, playing a crucial role in the synthesis of chiral amines and amino acids.^[^ ^17^, ^24^ ^]^ PLP forms a Schiff base with a lysine residue in the active site in the first half‐cycle of the reaction as part of a “ping‐pong bi‐bi” mechanism. The amino donor undergoes deamination to produce the donor product and pyridoxamine‐5′‐phosphate (PMP). As the amino acceptor is aminated, PMP releases the amino group, forming PLP and generating a new amine or amino acid.^[^ ^25^, ^26^ ^]^ TAs exhibit a broad substrate range, efficient cofactor regeneration, and excellent stereoselectivity, giving these enzymes advantages not seen in other enzyme classes.^[^ ^10^, ^20^, ^23^ ^]^ TAs play important roles in industry and can reduce the need for expensive homogeneous catalysts while lowering the high energy consumption associated with downstream purification processes.^[^ ^22^ ^]^

Amine transaminases (ATAs) are distinguished by their ability to aminate a wide range of ketones, aldehydes, and other carbonyl compounds, enabling the asymmetric synthesis of chiral amines from prochiral ketones without requiring a carboxyl group in the substrate. ATAs are one of the most versatile options for chiral amine synthesis.^[^ ^27^ ^]^ Among all ATAs, one particularly interesting enzyme is a transaminase from *Silicibacter pomeroyi* (*Sp‐*ATA, 3HMU) where a highly conserved arginine residue is typically responsible for binding the carboxyl group at the active site of transaminases. This flexible “flipping” Arg417 creates a cavity in the active site that can accommodate the amino/ketone group.^[^ ^27^, ^28^, ^29^, ^30^ ^]^ This flexibility gives *Sp‐*ATA a broad substrate scope. However, despite its excellent catalytic properties, *Sp‐*ATA has limitations in stability.^[^ ^31^ ^]^ Studies have shown that *Sp‐*ATA exhibits poor stability under extreme conditions such as high temperatures and organic solvents, and instability of the cofactor PLP.^[^ ^23^, ^32^ ^]^ Industrial processes demand enzymes that maintain stability and catalytic efficiency under nonphysiological conditions over time. Enzymes with poor stability tend to deactivate, requiring frequent replenishment and complex enzyme immobilization steps, which increase production costs.^[^ ^33^, ^34^ ^]^ Additionally, industrial biocatalysis frequently involves harsh conditions, such as high substrate concentrations, non‐aqueous systems, and extreme pH values,^[^ ^35^, ^36^ ^]^ making enzyme stability crucial for maintaining activity and preventing downtime.^[^ ^37^ ^]^ Thermostability is widely regarded as a key factor in enhancing overall enzyme stability, as it strengthens protein structure and improves resistance to extreme environments.^[^ ^38^ ^]^ Studies show that thermostable enzymes possess enhanced hydrogen bonding networks, hydrophobic cores, and disulfide bonds, making them more resilient under industrial conditions.^[^ ^39^ ^]^ Enhancing enzyme stability ensures that it maintains catalytic activity under these conditions, preventing downtime caused by enzyme deactivation, thus improving production efficiency. However, *Sp*‐ATA's catalytic activity diminishes significantly in these environments, restricting its industrial applicability.^[^ ^40^ ^]^

To overcome these limitations, we aim to improve the stability of *Sp‐*ATA. A primary objective in engineering transaminases is improving thermostability through various strategies, including charge–charge interactions, proline substitutions, and the stabilization of protein quaternary structures by preventing dimer dissociation.^[^ ^41^, ^42^, ^43^, ^44^, ^45^ ^]^ For instance, using B‐factor analysis in combination with the proline rule, Land et al. improved the thermostability of the amine transaminase from *Chromobacterium violaceum*.^[^ ^41^ ^]^ Cao et al. increased the thermostability and activity of a transaminase from *Aspergillus terreus* by identifying four surface‐exposed residues hypothesized to enhance thermal stability.^[^ ^44^ ^]^ Enzyme functions have been enhanced by directed evolution in the past, but it is challenging to predict the effects of combining individual stabilizing mutations.^[^ ^46^, ^47^, ^48^, ^49^ ^]^ Computational methods have also been applied, but these are often time‐consuming and require a detailed structure to generate training data sets.^[^ ^25^, ^50^ ^]^ Therefore, an alternative method using ancestral sequence reconstruction (ASR) has emerged as a potential solution,^[^ ^51^ ^]^ which leverages evolutionary history to identify residues critical for maintaining protein function. Although less frequently used, ASR has shown potential as a tool for engineering thermostable proteins.^[^ ^52^, ^53^, ^54^ ^]^ Previous studies have demonstrated that ASR can generate transaminases with broader substrate scope and significantly improved catalytic activity, with up to 20‐fold increases in efficiency compared to modern enzymes.^[^ ^55^ ^]^ Notably, ASR‐derived transaminases have exhibited enhanced conversion of industrial feedstocks such as 12‐amino‐dodecanoic acid, a key precursor for nylon‐12.^[^ ^52^ ^]^ Structural analyses of the reconstructed enzymes revealed that substrate preference was influenced by broader structural features beyond the active site, highlighting the potential of ASR as a powerful alternative to traditional enzyme engineering.^[^ ^55^ ^]^ Building on these findings, we propose to screen and reconstruct more stable ancestral enzymes from a gene library based on the amino acid sequences of *Sp*‐ATA. By expressing and characterizing some of these ancestral enzymes, we aim to validate whether ASR can be applied to enhance the stability of transaminases.

## 2 Results and Discussion

### 2.1 ASR of ATAs

**Figure 1:**
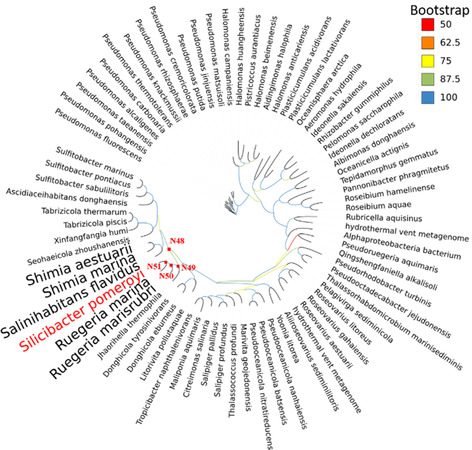
Phylogenetic tree of the 75 transaminase sequences selected from UniProtKB using BLAST with *Sp*‐ATA (PDB: 3HMU) as a query. Sequences were filtered by length (400–600 residues) and identity thresholds (90% for selection, 97% for redundancy reduction). The tree was constructed using IQ‐TREE with the JTT model and 1000 bootstrap replicates. Ancestral sequences were reconstructed with FastML, and the nodes of the same are marked with red dots. Branch colors represent bootstrap values.

The enzymes in our panel exhibit 60%–95% sequence identity with the benchmark enzyme, *Sp*‐ATA. All amino acid sequences of these enzymes are provided in the supplementary materials. To identify ancestral enzymes with potentially higher thermostability, we selected sequences from bacteria that thrive at temperatures above 40 °C for ASR. The resulting phylogenetic tree (**Figure** **1**) was analyzed, and marginal reconstruction algorithms with optimized likelihood scores were applied in FastML to generate ancestral proteins. Ancestral sequences were chosen from the four closest nodes to *Sp*‐ATA (from closest to farthest: N51, N50, N49, and N48) for further study. Among them, N48 and *Sp*‐ATA have the lowest sequence identity of only 87.1%. Moving toward the youngest ancestor, N51, the sequence identity increases: from N49 with 88.7% to N50 with 92.2% and N51 showing the highest sequence identity of 94.3% (**Table** **1**). The decision to reconstruct these specific ancestors is based on their evolutionary proximity to *Sp*‐ATA. By selecting these closer nodes, we aim to retain a similar substrate range to that of *Sp*‐ATA, as they are expected to exhibit more similar structural and functional characteristics compared to ancestors from more distant nodes within the phylogenetic tree. This assumption relies on the principle that genetic divergence accumulates over time, making more distant ancestors likely to show greater differences from the contemporary protein.^[^ ^56^ ^]^

**Table 1:** Sequence identity for the ancestors to *Sp*‐ATA with the root mean standard deviation (RMSD) of the ancestors aligned to *Sp*‐ATA.

Enzymes	Sequence identity [%]	RMSD [Å]
N48	87.1	0.90
N49	88.7	0.81
N50	92.2	0.57
N51	94.3	0.67

### 2.2 Kinetics and Substrate Range of the Ancestral Enzyme Variants

**Figure 2:**
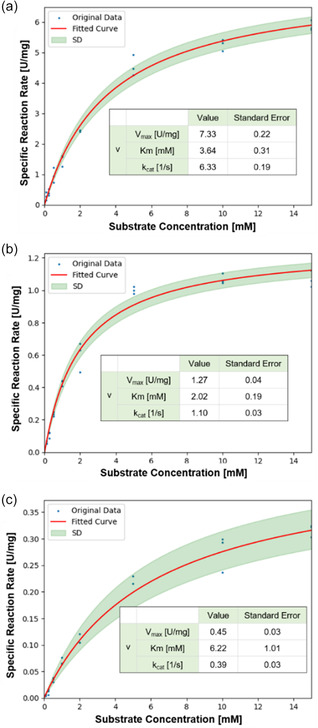
The Michaelis–Menten kinetic constants of a) *Sp*‐ATA, b) N49, and c) N50. Kinetic parameters were determined using the acetophenone assay with PEA as the amine donor and Na‐pyruvate as the amino acceptor. Reactions were performed in triplicate with six PEA concentrations (0.1–15 mM) and initiated by adding 10 μg enzyme with 5 mM PLP. Acetophenone formation was monitored at 245 nm, and standard deviations were typically <20%.

After successfully expressing and purifying soluble ancestral proteins (Figure S2, Supporting Information), further assays were conducted to determine the optimal pH for the ancestral enzyme variants. The activities of the ancestral enzymes N48, N49, N50, and N51 were tested in 50 mM Tris‐HCl buffer at pH 7.5, 4‐(2‐hydroxyethyl)‐1‐piperazineethanesulfonic acid (HEPES) buffer at pH 8.2, and *N*‐cyclohexyl‐2‐aminoethanesulfonic acid (CHES) buffer at pH 9.0. The acetophenone assay was used to measure activity and confirm the selectivity of the selected ancestral enzymes. To ensure the functional relevance of the selected sequences, we experimentally tested the activity of the reconstructed ancestral enzymes. N51, N50, and N49 exhibited confirmed *S*‐selective transaminase activity (Figure S3, Supporting Information). This experimental verification mitigates concerns about misclassification due to automatic annotation.^[^ ^32^ ^]^ Additionally, structural analysis based on homology modeling described in the following section further supports the expected activity and selectivity of these enzymes. The results showed that all ancestral enzymes, except N48 (which, despite being soluble, exhibited no activity, likely due to improper folding or active site structural constraints), along with the wild‐type *Sp*‐ATA, performed best in the pH 9.0 CHES buffer (Figure S3, Supporting Information), consistent with previous results.^[^ ^30^ ^]^ As for N51, although its phylogenetic position suggests activity similar to the wild type, its significantly lower activity may be attributed to factors beyond sequence similarity, such as the need for post‐translational modifications,^[^ ^57^ ^]^ folding issues, or molecular interactions. N51 was excluded from further analyses due to its significantly lower activity compared to *Sp‐*ATA wild type. To compare the catalytic efficiency of ancestral enzymes with *Sp*‐ATA, we measured the kinetic parameters (V_max_, *K*_m_, and *k*_cat_) of *Sp*‐ATA, N49, and N50 with the substrate (*S*)‐PEA. As shown in **Figure** **2**, the ancestral enzymes displayed decreased catalytic efficiency relative to *Sp*‐ATA, which may indicate either a reduction in overall catalytic ability or that (*S*)‐PEA is not their optimal substrate. A plausible explanation could be that these thermostable enzymes tend to have a more rigid structure at room temperature, requiring higher temperatures for optimal activity.^[^ ^56^, ^58^ ^]^ Additionally, evolutionary changes in the ancestral proteins may have led to the loss of activity, particularly in N48, which is the most ancestral of the four proteins. While the phylogenetic tree provides insights into evolutionary relationships, it does not always predict functional outcomes, especially when unexpected structural or dynamic changes occur.^[^ ^59^ ^]^ Further modifications in the ancestral proteins might contribute to reduced activity, potentially due to new interactions between different regions (e.g., the active site), which could enhance stability but compromise activity. These findings are consistent with the broader goal of engineering enzymes for enhanced thermal stability, where improved stability may come at the cost of reduced catalytic activity due to structural rigidity or altered active site interactions.

**Figure 3:**
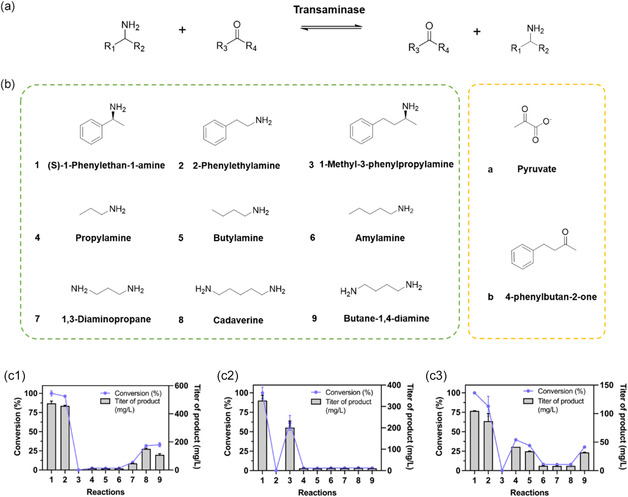
Determination of substrates scope. a) Schematic diagram of the transaminase reaction. b) Amino donors (**1–9**) and amino acceptors (**a, b**) used in the catalytic reaction. c) Catalytic performance of *Sp*‐ATA (c1), N49 (c2), and N50 (c3) on various substrates. Titer of product (mg L^−1^) represents the concentration of the target compound produced. Reactions were conducted at 30 °C in 50 mM CHES buffer (pH 9.0) with 10 mM amine acceptors (a, b), 5 mM amine donors (**1–9**), 1 mM PLP, and 0.5 mg purified enzyme. Product titers (mg L^−1^) were quantified via HPLC after 20 h. All reactions were conducted in triplicates.

**Figure 4:**
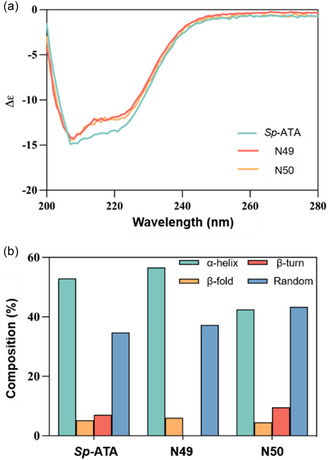
Conformation changes of ancestral enzymes compared to *Sp*‐ATA. a) Circular dichroism spectra of *Sp*‐ATA, N49, and N50 at 200–280 nm using a Chirascan CD spectrometer. Protein samples (0.1 mM) were measured in a 1 mm path‐length cuvette. Spectra were averaged over five scans, baseline‐corrected, and plotted using Prism GraphPad. b) Corresponding secondary structure composition.

The activities of the ancestral variants were then screened against nine amine donors, which included three aromatics (**Figure** **3**b, **1**–**3**), three aliphatic monoamines (Figure 3b, **4–6**), and three aliphatic diamines (Figure 3b, **7–9**), all at 5 mM concentration. For the aromatic amine donors, sodium pyruvate served as the amine acceptor. For the aliphatic donors, the aromatic acceptor 4‐phenyl‐2‐butanone (PEB) was used to allow detection with a high‐performance liquid chromatography (HPLC) UV detector (Figure 3b). Results displayed in Figure 3c indicate the product conversion rates with substrates **2–9** (the reactions numbered as the applied substrates) relative to (*S*)‐PEA. Compared to *Sp*‐ATA, N49 showed an interesting loss of conversion for **2** but a significant increase for **3**. This shift in substrate preference may be attributed to structural changes in the active site, particularly the repositioning of key amino acids influencing substrate binding. Specifically, the outward‐facing F92 (F94) side chain in N49 may increase the space in the binding pocket, making it easier for larger substrates like substrate **3** to enter. In contrast, substrate **2**, which is smaller, may require a tighter binding pocket, and the altered pocket structure in N49 may reduce or eliminate its binding affinity. Additionally, N49 exhibited nearly no reactivity with reactions **7**–**9** compared to *Sp*‐ATA. N50 demonstrated alternative substrate selectivity, with enhanced activity for reactions **4** and **5** and markedly reduced activity for **7** and **8**. This shift suggests that changes in the active site architecture may have optimized N50 for binding smaller aliphatic amines. Notably, in N50, the inward‐facing R417 (R426) may cause the active pocket to be more suited for smaller, straight‐chain amine substrates, enhancing the binding affinity for substrates like propylamine and butylamine. This phenomenon has been confirmed in previous studies, where thermostable enzymes exhibited superior activity at higher temperatures compared to their less thermostable counterparts. Additionally, sequence and structural changes in the ancestral variants may also contribute to a decrease in activity.^[^ ^58^, ^60^ ^]^ We propose that structural changes in the active site have led to alterations in the substrate range of the ancestral enzymes. To better understand how conformational change affects the substrate domain and activity of N49 and N50, we analyzed the secondary structure of *Sp*‐ATA, N49, and N50 using circular dichroism spectroscopy (**Figure** **4**a). Compared to *Sp*‐ATA, both N49 and N50 show distinct differences in the composition of their secondary structure elements (α‐helix, β‐sheet, β‐turn, and random coils) (Figure 4b). These structural differences may significantly impact the enzyme's substrate domain and activity, contributing to the observed shifts in substrate preference.

### 2.3 Computational Analysis of Structural Differences and Active Site Conservation in Ancestral Proteins

**Figure 5:**
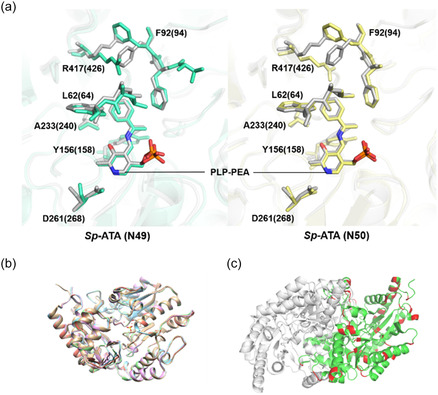
Structural comparison of *Sp*‐ATA and the ancestral enzymes. a) Docking simulations of the ancestral enzyme and the PLP‐PEA ligand complex, the structures of *Sp*‐ATA, N49, and N50 are shown in gray, cyan, and blue, respectively, with the corresponding PLP‐PEA ligand in matching colors. Residue numbers in parentheses represent the ancestral enzymes, while those without parentheses represent *Sp*‐ATA. b) Alignment of homology‐modeled ancestral structures with *Sp*‐ATA. c) Dimeric structure of *Sp*‐ATA, with red regions highlighting sequence differences among the four ancestral enzymes, N48, N49, N50, and N51. Homology models were built in YASARA using hm_build.mcr with a dimeric state. Ancestral sequences and templates were identified via PSI‐BLAST, and 25 models per ancestor were generated and refined. Hybrid structures were analyzed, and molecular docking used these models alongside the *Sp*‐ATA crystal structure (PDB: 3HMU) with PLP (PDB: 3FCR). Structures were aligned in YASARA, with SO_4_ and obstructive H_2_O removed. PLP protonation was adjusted, a 5 Å simulation cell was set, and energy minimization was performed using the AMBER03 force field. Fifty docking simulations were run with AutodockLGA to evaluate binding poses. All images were generated using PyMOL.

To visualize the impact of structural differences in the active site, homology modeling was employed. Alignment of the homology‐modeled structures for the ancestral proteins with the wild‐type *Sp*‐ATA showed a high degree of similarity (**Figure** **5**b). For a more precise analysis, docking simulations with PLP‐PEA were conducted to assess the active site, yielding an interesting observation. As reported by Steffen–Munsberg et al.^[^ ^28^ ^]^ the residues forming the active site—including Lys258, responsible for forming an internal aldimine with PLP, Asp259, which stabilizes and orients PLP during catalysis, and Arg417, which plays a role in substrate binding and specificity—are conserved across all four ancestral proteins as well as the wild‐type *Sp*‐ATA (Figure 5a). From a structural perspective, this suggests that the observed changes in activity and stability in the ancestral enzymes may arise from alterations in regions outside the active site. However, compared to *Sp*‐ATA, the residue numbering in the ancestral proteins has changed, although their positions are nearly similar. The numbers in parentheses correspond to the ancestral proteins, while those outside correspond to the *Sp*‐ATA wild type. Additionally, further analysis of the ancestral sequences through multiple sequence alignment (MSA) (Figure S2, Supporting Information) revealed amino acid insertions in certain regions, which are illustrated in one subunit of N49 (Figure S3, Supporting Information) and are normal occurrences in ancestral enzymes. Furthermore, in the same study on *Sp*‐ATA, Steffen–Munsberg et al.^[^ ^28^ ^]^ highlighted the key role of the “flipping” arginine residue (R417), which is crucial for dual substrate recognition. When R417 points towards the PLP, it can accept α‐carboxylic substrates. Conversely, when the arginine flips upwards in the active site, it creates a larger binding pocket, enabling the binding of larger substrates, such as (*S*)‐PEA. When each ancestral protein is aligned with *Sp*‐ATA, this arginine adopts a more extended conformation towards PLP‐PEA across all ancestral proteins compared to its conformation in *Sp*‐ATA (Figure 5a). This may suggest that (*S*)‐PEA is less favored as an amino donor for these enzymes.

**Figure 6:**
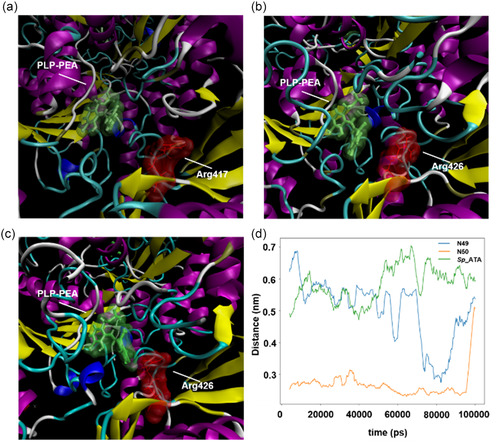
MD simulation of the docked PLP‐PEA complex with *Sp*‐ATA, N49, and N50. a–c) Structural visualization of the PLP‐PEA complex (green surface) and the “flipping” arginine (red surface) in a) *Sp*‐ATA, b) N49, and c) N50. In *Sp*‐ATA, the arginine extends outward, positioned farthest from the PLP‐PEA. In N49, it partially flips inward, while in N50, it adopts the most inward conformation. d) The shortest pairwise distance between the “flipping” arginine and PLP‐PEA shows a trend consistent with the structures: *Sp*‐ATA has the largest distance, followed by N49, with N50 having the shortest. MD simulation details: Simulations were performed using GROMACS 2024.2 with the AMBER99SB‐ILDN force field for proteins and TIP3P for water. Ligands were parametrized using GRAFF via ACPYPE. Systems were solvated in a triclinic box (≈1500 nm^3^), neutralized with Cl^−^ ions, minimized, equilibrated (NVT/NPT, 200 ps at 300 K), and subjected to a 100 ns production run. Visualization was performed with VMD, and quantitative analysis used GROMACS gmx pairdist, plotting results in Python.

To further validate the docking results and our hypothesis, molecular dynamics (MD) simulations were performed on the docked structures of *S*p‐ATA, N49, and N50 with PLP‐PEA. The simulations revealed distinct conformational differences in the “flipping” arginine residue, further supporting our observations. As shown in **Figure** **6**, in *Sp*‐ATA, the “flipping” arginine extends outward, positioned farther from the PLP‐PEA ligand. In N49, the arginine exhibits partial inward flipping, while in N50, it adopts the most pronounced inward conformation. Additionally, analysis of the shortest pairwise distance between the flipping arginine and PLP‐PEA (Figure 6d) corroborates these structural differences: *Sp*‐ATA exhibits the largest distance, followed by N49, while N50 has the shortest distance. These findings reinforce our hypothesis that the altered flipping behavior of this arginine residue in ancestral proteins may influence their substrate specificity and catalytic properties.

### 2.4 The Ancestral Variants Exhibit a Significant Increase in Thermostability

**Figure 7:**
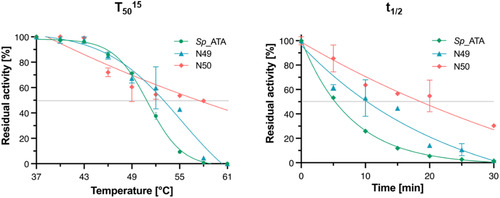
The thermostability of *Sp*‐ATA and its ancestral variants (N49, N50, and N51) was evaluated by determining the half‐inactivation temperature (T_50_^15^) and the half‐life (t_1/2_). T_50_^15^ denotes the temperature at which enzyme activity is reduced to 50% after 15 min of heat exposure, whereas the t_1/2_ represents the time required for enzyme activity to decrease to 50% at 55 °C. The enzymes were incubated at 55 °C for up to 60 min, followed by cooling, and their activity was measured at 22 °C. An exponential decay model in GraphPad Prism 10 was used to analyze the data and determine the point of 50% activity loss. Furthermore, the enzymes were subjected to 15‐min incubations at temperatures ranging from 37 to 61 °C and then cooled before assessing activity. All experiments were conducted in triplicate.

Research has frequently highlighted the greater stability of ancestral enzymes compared to their modern counterparts. To investigate this stability, we measured half‐inactivation temperature (T_50_^15^) and the half‐life (t_1/2_) of the ancestral enzymes (**Figure** **7**). For instance, N49 showed a T_50_^15^ of 54.9 °C, an increase of about 4 °C over the T_50_^15^ of the wild‐type *Sp*‐ATA, which is 51 °C (Figure 7a,c). Additionally, the half‐life of N49 extended twofold, from 5 to 10.3 min (Figure 7d,f). N50, in comparison, exhibited even more significant stability enhancements, with a T_50_^15^ of 6 °C increase and a half‐life extended by 3.7 times to 18.6 min (Figure 7b,e). These results suggest the potential of ancestral reconstruction as an effective strategy for enhancing enzyme stability. To address concerns regarding potential renaturation effects after heat treatment, we ensured strict consistency in temperature control and conducted activity assays immediately after cooling. While renaturation could theoretically influence measured activity, the observed significant increase in thermostability among ancestral enzymes strongly suggests an inherent stabilization effect rather than a transient recovery. The marked increases in T_50_^15^ and half‐life highlight the robustness of the enzyme variants under thermal stress, indicating that evolutionary adaptations retained in ancestral enzymes can confer substantial stability advantages. This stability may stem from structural features that enhance robustness at elevated temperatures, a hypothesis that is worth further investigation to identify specific structural contributors to this enhanced stability. The superior stability of N49 and N50 makes them particularly promising for industrial processes, thus broadening the practical applications of transaminases. While ancestral reconstruction offers stability benefits, a well‐known limitation of the ASR method is the challenge of linking structural modifications to functional outcomes.^[^ ^61^ ^]^ Numerous amino acid substitutions are present in the ancestral proteins, scattered across different protein regions—particularly in loops and α‐helices (Figure 5c and S1, Supporting Information). Although pinpointing the exact contribution of each substitution remains challenging, the distribution of these substitutions may guide further rational design, focusing on regions that could improve the thermal stability of other transaminases.

### 2.5 Organic Solvents Stability

**Figure 8:**
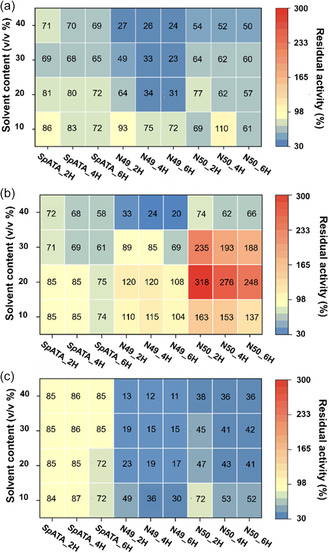
Enzyme tolerance to different concentrations of a) MeOH, b) DMSO, and c) acetonitrile after incubation for 2, 4, and 6 h at 25 °C. Purified enzymes were incubated in a mixture of organic solvent (0%, 10%, 20%, 30%, and 40% v v^−1^) and CHES buffer. Residual enzyme activity was then determined using an acetophenone assay and measured by HPLC. Reactions without enzymes as blanks, while those without organic solvents were controls. All reactions were performed in triplicates.

To determine the enzyme tolerance to the reaction media, the wild type, N49, and N50 were incubated in 10, 20, 30, and 40% concentrations of MeOH, dimethyl sulfoxide (DMSO), and acetonitrile for 2, 4, and 6 h, followed by a 20 h reaction to measure the acetophenone yield (**Figure** **8**). Results showed that the presence of DMSO significantly enhanced the activity of ancestral enzymes N49 and N50, a phenomenon not observed in the wild‐type *Sp*‐ATA (Figure 8b). Specifically, N49 displayed an increase in activity in 10%–30% DMSO, with activity more than triple after a 2‐h incubation in 20% DMSO. N50 also showed slight activity enhancements in 10% and 20% DMSO. However, both ancestral enzymes experienced activity loss in 40% of DMSO. In contrast, the wild‐type *Sp*‐ATA performed poorly in all DMSO concentrations, retaining only about half of its activity in 40% DMSO. In the presence of MeOH, the wild‐type *Sp*‐ATA exhibited only a minor decrease in catalytic activity with increasing concentration and incubation time (Figure 8a). For instance, after a 6‐h incubation in 40% MeOH, *Sp*‐ATA retained around 70% of its activity. However, N49 showed significant declines in activity with increasing MeOH concentration and incubation time. After 6 h in 10% MeOH, N49 retained 72% of its activity, but this dropped to 31% in 20% MeOH and only 24% in 40% MeOH. In acetonitrile, the ancestral enzymes showed the poorest tolerance, while the wild‐type *Sp*‐ATA displayed some degree of resistance (Figure 8c). This variation in enzyme stability and activity across different solvents can be attributed to how the solvents interact with the protein. Acetonitrile is a polar aprotic solvent, which can destabilize the enzyme's native conformation, causing protein unfolding and reduced activity.^[^ ^62^ ^]^ In contrast, DMSO, also a polar aprotic solvent with strong hydrogen bond‐accepting ability,^[^ ^63^ ^]^ possibly stabilizes the enzyme's secondary and tertiary structures by preserving essential surface hydration through its S=O structure, thereby enhancing enzyme stability and activity.^[^ ^64^ ^]^ Overall, this experiment reveals differences in reaction medium tolerance between the ancestral enzymes and the wild‐type *Sp*‐ATA, which provides understanding and insights for developing industrial enzymes.

## 3 Conclusion

This study utilized ASR with the amino acid sequence of *Sp*‐ATA as a template. Four ancestral enzymes (N48, N49, N50, and N51) were selected from branches closest to *Sp*‐ATA for further investigation. The study showed that all four ancestral enzymes were successfully expressed in *Escherichia coli (E. coli)*. Among these, N49 and N50 exhibited promising catalytic activity and substrate specificity distinct from *Sp*‐ATA, whereas N48 showed no activity with the amino donor (*S*)‐PEA, and N51 had negligible activity. The ancestral enzymes N49 and N50 also demonstrated improved thermal stability with an increase in T_50_^15^ values by 3.9 and 6 °C, respectively, relative to *Sp*‐ATA, and their half‐lives (t_1/2_) at 55 °C were extended 2.06 to 3.72 times before reaching inactivation. Furthermore, we compared solvent tolerance across the ancestral enzymes and found that both N49 and N50 displayed a preference for DMSO. In lower concentrations of DMSO, they exhibited significantly enhanced catalytic activity. However, their tolerance to methanol and acetonitrile was lower than that of the wild‐type enzyme. These findings provide valuable insights into enzyme adaptability to specific solvents and strategies for enhancing enzyme thermal stability, thereby supporting the use of biocatalysts in industrial applications. Nonetheless, a deeper understanding of enzyme structure will be crucial to precisely control modifications, preserving high activity and substrate specificity while boosting stability.

## 4 Experimental Section

4.1

4.1.1

##### Materials

All chemicals of analytical or higher grade were purchased from Sigma–Aldrich Sweden. Yeast extract, tryptone, agar and ampicillin were purchased from Merck Sweden. Agarose and NaCl were obtained from VWR Sweden. The Ni/NTA His Bind resin for His‐trap column was purchased from Merck Sweden. The PD10 column was purchased from Cytiva Sweden.

##### Medium, Plasmids, and Strains

The lysogeny broth medium (LB, 10 g L^−1^ NaCl, 10 g L^−1^ tryptone, and 5 g L^−1^ yeast extract, pH 7.0) was utilized for growing *E. coli* cultures. Protein expression was conducted in terrific broth (TB, 16 g L^−1^ tryptone, 10 g L^−1^ yeast extract, NaCl, pH 7.0). Agar was added to a final concentration of 1.5% to prepare solid medium. 5 g L^−1^ Ampicillin was added to the medium at a concentration of 100 μg mL^−1^. The strains *E. coli* XL1‐Blue and BL21(DE3) were used for plasmid amplification and expression respectively.

##### Sequence Collection

The amino acid sequence of *Sp‐*ATA (PDB: 3HMU) was used as a query to retrieve homologous transaminase sequences. The search was conducted against the UniProtKB database (https://www.uniprot.org/) using the basic local alignment search tool (BLAST) with default parameters. Unique sequences were identified and cleaned using the CD‐HIT^[^ ^65^ ^]^ program with a 90% sequence identity threshold to ensure nonredundant data. The resulting sequences were manually reviewed to remove false positives, including those shorter than 400 or longer than 600 residues, as well as sequences with clear duplicates or missing fragments. Further data cleaning was performed by removing sequences with more than 97% identity to *Sp*‐ATA. Furthermore, an automated script was created using Python, incorporating Matplotlib 3.7.1,^[^ ^66^ ^]^ NumPy 1.26.2,^[^ ^67^ ^]^ and Pandas 1.5.3 module (Zenodo DOI: 10.5281/zenodo.13819579). The script was used to filter out sequences from microorganisms with optimal growth temperatures below 40 °C. Furthermore, we validated the presence of the PLP‐dependent transferase domain using InterPro annotations,^[^ ^68^ ^]^ confirming that all sequences belong to the same structural and phylogenetic family as *Sp*‐ATA. These filtering criteria minimized the risk of including unrelated or nonfunctional sequences and ensured a robust foundation for MSA and phylogenetic tree construction. The final dataset contained 75 sequences.

##### MSA and Phylogenetic Analysis

A multiple sequence alignment (MSA) of the dataset was generated using MAFFT^[^ ^69^ ^]^ with the FFT‐NS‐I ×1000 algorithm (fast fourier transform‐based progressive alignment with iterative refinement), which is known for its high alignment accuracy and efficiency in handling large‐scale datasets. This advanced alignment strategy incorporates FFT and iterative optimization to achieve high computational efficiency while maintaining alignment precision. Given the scale of the dataset and the need for both speed and accuracy, the FFT‐NS‐I ×1000 algorithm was particularly suitable for the analysis of this work. Multiple runs of the algorithm were performed to obtain consistent results, which confirmed the stability of the outcome. Additionally, the alignment results were compared with those from ClustalW, yielding similar outcomes and further validating the accuracy of the FFT‐NS‐I ×1000 algorithm. Gaps within the aligned sequences were removed, and a maximum likelihood (ML) phylogenetic tree was constructed in IQ‐TREE using the Jones‐Taylor‐Thornton substitution (JTT) model with 1000 bootstrap replicates. ASR was performed with FastML,^[^ ^70^ ^]^ and the ancestral tree was visualized in iTOL.^[^ ^71^ ^]^ Homology modeling of the ancestral sequences was carried out in YASARA (version 23.12.24)^[^ ^72^ ^]^ and visualized using PyMOL (version 4.6.0).^[^ ^73^ ^]^

##### Expression and Purification

The genes encoding ancestral enzymes were synthesized and cloned into pET22b (+) with ampicillin resistance by Twist Bioscience (San Francisco, USA). The plasmids were transformed into *E. coli* XL1‐Blue. For *Sp*‐ATA, the gene was previously cloned into pET22b (+) with ampicillin resistance. For overexpression, plasmids pET22b‐*Sp‐ATA*, pET22b‐*N48*, pET22b‐*N49*, pET22b‐*N50*, and pET22b‐*N51* were transformed into *E. coli* BL21(DE3). Colonies were grown in LB with 50 μg mL^−1^ ampicillin at 37 °C, 220 RPM. Protein expression was induced with 0.2 mM IPTG at OD_600_ = 0.6, followed by incubation at 20 °C, 200 RPM for 20 h. Cells were harvested (4,000 × g, 20 min, 4 °C), resuspended (1:1.5, w v^−1^) in lysis buffer, lysed via sonication, and centrifuged (15 000 × g, 20 min, 4 °C) to collect the supernatant. For purification, 1 mL of Ni‐NTA agarose was loaded into a column, and the cell lysate was allowed to pass the column. The column was then washed with washing buffers 1 and 2 (50 mM NaCl, and 20 mM or 50 mM imidazole) to remove contaminants. Target protein was eluted with 0.5 mL of buffer with 50 mM NaCl, and 300 mM imidazole. Desalting was performed using a PD‐10 column. Purified proteins were analyzed on 10% sodium dodecyl sulfate polyacrylamide gel electrophoresis (SDS‐PAGE, Bio‐Rad TGX gel), run at 220 V for 30 min in 1× SDS‐Tris buffer. SeeBlue Plus2 Pre‐stained Protein Standard was used as standard and Sigma Laemmli 2× Concentrate were used as sample buffers (20 μL per sample). For dimer formation, enzymes were incubated with 5 mM PLP at 4°C overnight. Then, excess PLP was removed using PD10 column with either 50 mM HEPES buffer (pH 8.2) or 50 mM CHES buffer (pH 9.0).

##### Enzyme Activity Assay

The conversion of (*S*)‐1‐phenylethylamine (PEA) to acetophenone^[^ ^74^ ^]^ was monitored at 245 nm using an extinction coefficient of 12 mM^−^^1^ cm^−^^1^. Acetophenone formation was measured every 5 s over a 3‐min period with a Cary50 ultraviolet (UV)‐visible spectrophotometer, using 1 mL UV cuvettes. The assay was conducted at 22 °C, with a typical reaction mixture (1 mL) consisting of 5–50 μg of an enzyme, 2.5 mM PEA, and 2.5 mM sodium pyruvate in different buffers. To determine the optimal pH of the ancestral enzyme, the reactions were carried out in 50 mM Tris‐HCl buffer at pH 6.0 and pH 7.5, in HEPES buffer at pH 8.2, and in CHES buffer at pH 9.0. All measurements were performed in triplicate, and protein samples were incubated at 22 °C for 1 h before the assay.

##### Kinetic Analysis

Kinetic assays were performed in triplicate using a microplate reader. The acetophenone assay was performed to assess the kinetic analysis. The PEA was an amine donor, and Na‐pyruvate was used as an amino acceptor. To obtain the Michaelis–Menten constant (K^app^_M_), six different concentrations of (*S*)‐PEA, ranging from 0.1 to 15 mM, were used. Reactions were started by adding 10 μg enzyme with 5 mM PLP. The generation of acetophenone followed over time by measuring the 245 nm absorbance. The standard deviations for the determined kinetic parameters were typically <20%.

##### Secondary Structure Assessment

Circular dichroism (CD) spectra were recorded using a Chirascan CD spectrometer. Protein samples were prepared at a concentration of 0.1 in 50 mM CHES buffer at pH 9.0, with a sample volume of 400 μL. The measurements were carried out in a 1 mm path‐length cuvette. The UV range spanned from 200 to 280 nm, using a quartz cell with a 0.2 mm path length. The scan speed was set to 50 nm min^−1^, with a response time of 1 s, and a total of five scans were accumulated. Baseline correction was applied to all spectra, and the data was subsequently plotted using Prism GraphPad (version 10.2).

##### Thermostability Determination

Both the half‐inactivation temperature (T_50_^15^) and half‐life (t_1/2_) values characterize the thermostability of the enzyme. T_50_^15^ refers to the temperature at which the enzyme activity was reduced to half of the original activity after heat treatment at a continuous temperature for 15 min. The enzyme solution was incubated for 15 min at temperatures of 37, 40, 43, 46, 49, 52, 55, 58, and 61 °C and then cooled on ice for 10 min. Using the Levenberg–Marquardt iterative algorithm, the data were fitted to a four‐parameter Boltzmann sigmoidal function. The formula is presented in Equation (1).

(1) $$R\;=\;A\;+\;\left(B\;-A\right)/\left(1\;+e^{\left(T_{m}\;-\;T\right)/C}\right)$$

where *A* and *B* represent pretransition and post‐transitional residual activity, respectively, at temperature *T*. *C* is the slope factor, controlling the steepness of the curve or the temperature sensitivity. *T* is the current temperature. *T*_0_ is the temperature in the absence of treatment. In the absence of treatment, *T*_m_ = *T*_0_, when there is no treatment.

Similarly, t_1/2_ is defined as the time when the residual activity of *Sp*‐ATA and its ancestors was reduced to 50% of its original activity. For the study, we measured the t_1/2_ at 55 °C. The purified *Sp*‐ATA, N49, N50, and N51 variants were incubated for 0–60 min at 55 °C and then cooled on ice for 10 min. The enzyme activity test was performed at 22 °C for 3 min with a spectrophotometer under 245 nm. The data was fitted with an exponential function model: [Y = (Y_0_ ‐ Plateau) * exp(‐K*X) + Plateau] (Y_0_, Y ‐value at X = 0, same unites as Y; Plateau, Y ‐value at infinite time, same units as Y; and K, rate constant, reciprocal of X ‐axis units) by nonlinear regression using GraphPad Prism 10 (GraphPad Software Inc., La Jolla, CA, USA) and then the 50% of relative enzyme activity were determined.

##### Substrate Scope Screening

To evaluate the substrate scope of ancestral enzymes compared to *Sp*‐ATA, nine amine donors were selected. The substrate spectrum of the ancestral enzymes was determined at 30 °C in a 0.5 mL reaction mixture containing 50 mM CHES buffer (pH 9.0), 10 mM acceptor (sodium pyruvate and PEB for R4‐9), the nine amine donors (Figure 3b), 1 mM PLP, and 0.5 mg of purified enzyme. The conversion of (*S*)‐PEA (following acetylation) to acetophenone was measured after 20 h via HPLC, using a flow rate of 1 mL min^−1^, a mobile phase of 20/80% v v^−1^ acetonitrile and water with 1% trifluoroacetic acid, and detection at 245 nm.

##### Solvent Tolerance

The impact of different concentrations of water‐miscible organic solvents (0%, 10%, 20%, 30%, and 40% v v^−1^), viz., DMSO, methanol, and acetonitrile, on enzyme activity was assessed by incubating purified enzymes in a mixture of organic solvent and CHES buffer for 2, 4, and 6 h at 25 °C. Residual enzyme activity was then determined using an acetophenone assay and measured by HPLC. A reaction without enzyme served as the blank, while a reaction without organic solvent was used as the control. All reactions were performed in triplicates.

##### Homology Modeling and Molecular Docking Simulations

The homology modeling in YASARA used the macro hm_build.mcr, modifying the oligo state from 4 to 2 for dimeric structures. It identified ancestral sequences and templates through PSI‐BLAST. After secondary structure predictions, 25 models per ancestor were built and refined in two steps. The best models were combined into hybrid structures for final analysis. Molecular docking simulations utilized the homology‐modeled structures and crystal structure 3HMU for *Sp*‐ATA, incorporating the essential cofactor PLP from PDB: 3FCR. Structures were aligned in YASARA, with SO_4_ and obstructive H_2_O molecules removed. The PLP protonation state was adjusted, and a simulation cell extending 5 Å was defined and filled with water. The AMBER03 force field was used for energy minimization. Pro‐(*S*)‐quinonoid complexes of PEA (PLP_PEA) were constructed, maintaining specific dihedral angles. Fifty independent docking simulations were executed with dock_run.mcr and AutodockLGA, evaluating binding poses and modes for PLP_PEA across structures.

##### MD Simulation

MD simulations were performed on the docked structures of *Sp*‐ATA, N49, and N50, each complexed with PLP_PEA and PLP at the dimer active site. All simulations were conducted using GROMACS 2024.2 (Zenodo DOI: 10.5281/zenodo.11148655). The AMBER99SB‐ILDN force field was used to parametrize the protein, while the TIP3P was employed for water. Ligands were parametrized using the GRAFF force field^[^ ^75^, ^76^ ^]^ via ACPYPE.^[^ ^77^ ^]^ Each system was placed in a triclinic periodic box with a volume of ≈1500 nm^3^, filled with TIP3P water molecules, and neutralized with Cl^−^ ions. Energy minimization was performed using the steepest descent algorithm for 50 000 steps or until convergence at –1000 kJ nm mol^−1^. The systems were equilibrated under NVT (constant volume and temperature equilibration run), followed by NPT (constant pressure and temperature equilibration run), for 200 ps each at 300 K. A 100 ns production run was then carried out, and the resulting trajectories were analyzed. The VMD program^[^ ^78^ ^]^ was used to visualize the docked substrate and the “flipping” arginine. Quantitative analysis was conducted using the GROMACS gmx pairdist function, and the results were plotted using Python 3.9.16 with Matplotlib 3.7.1.

## Conflict of Interest

The authors declare no conflict of interest.

## Supporting information

Supplementary Material

## Data Availability

The data that support the findings of this study are available in the supplementary material of this article.
