# Supplementary material for: Exploring the Stability and Substrate Profile of Transaminase from Silicibacter pomeroyi with Ancestral Sequence Reconstruction
Source: Chembiochem. 2025 May 30;26(13):e202500155. doi: 10.1002/cbic.202500155 (PMC12247033; doi:10.1002/cbic.202500155)
Supplement: Supplementary file 1 — Supplementary Material [file CBIC-26-e202500155-s001.pdf]

Exploring the Stability and Substrate Profile of Transaminase from *Silicibacter pomeroyi* with Ancestral Sequence Reconstruction

Luyao Zhao, [a] Bhu-Bhud Thongrakon, [a] Trishnamoni Gautom, [a] Viktor Sahlberg, [a] and Per Berglund*[a]

[a] Department of Industrial Biotechnology, School of Engineering Sciences in Chemistry, Biotechnology and Health. KTH Royal Institute of Technology, Stockholm, Sweden
E-mail: perbe@kth.se

Supporting Information

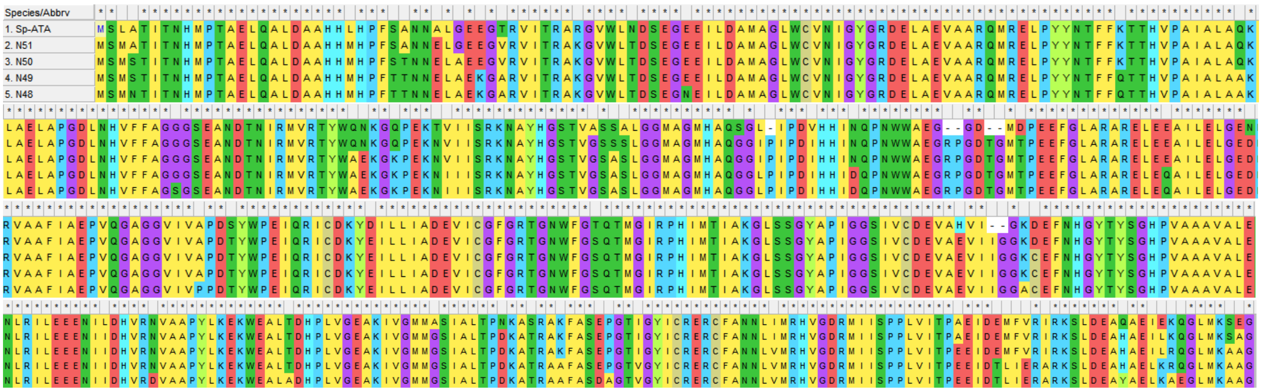

Figure s1 Amino acid sequences alignment of *Sp*-ATA, N48, N49, N50 and N51.

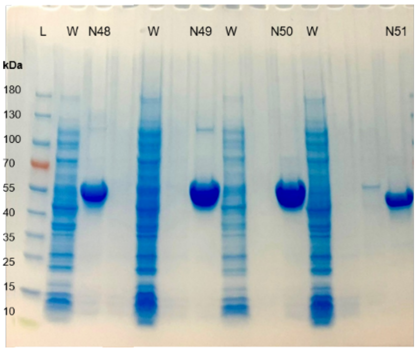

Figure s2 SDS-PAGE analysis of the ancestral enzymes. L: standard protein ladder; W: washing buffer collection; lanes N48-N51: purified protein samples.

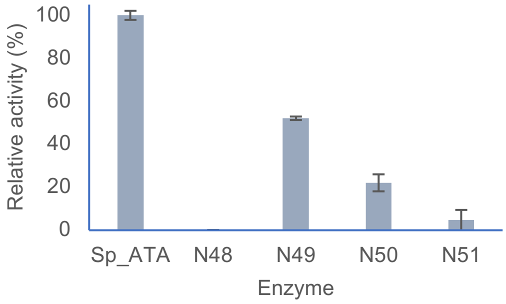

Figure s3 Specific activity of ancestral enzymes compared to *Sp*-ATA. The conversion of (*S*)-1-phenylethylamine to acetophenone was monitored at 245 nm using an extinction coefficient of 12 mM^{-1} cm^{-1}. Acetophenone formation was measured every 5 seconds over a 3-minute period using a Cary50 UV/Vis spectrophotometer with 1 mL UV cuvettes. The assay was performed at 22 °C, with each reaction mixture (1 mL) containing 5-50 µg of enzyme, 2.5 mM (*S*)-1-phenylethylamine, and 2.5 mM sodium pyruvate in various buffers.

Enzyme sequences:
>*Sp*-ATA
MSLATITNHMPTAELQALDAAHHLHPFSANNALGEEGTRVITRARGVWLNDSEGEEILDAMAGLWCVNIGYGRDELAEVAARQMRELPYYNTFFKTTHVPAIALAQK
LAELAPGDLNHVFFAGGGSEANDTNIRMVRTYWQNKGQPEKTVIISRKNAYHGSTVASSALGGMAGMHAQSGLIPDVHHINQPNWWAEGGDMDPEEFGLARAREL
EEAILELGENRVAAFIAEPVQGAGGVIVAPDSYWPEIQRICDKYDILLIADEVICGFGRTGNWFGTQTMGIRPHIMTIAKGLSSGYAPIGGSIVCDEVAHVIGKDEFNHGY
TYSGHPVAAAVALENLRILEEENILDHVRNVAAPYLKEKWEALTDHPLVGEAKIVGMMASIALTPNKASRAKFASEPGTIGYICRERCFANNLIMRHVGDRMIISPPLVIT
PAEIDEM>N51
MSMATITNHMPTAELQALDAAHHMHPFSANNELGEEGVRVITRAKGVWLTDSEGEEILDAMAGLWCVNIGYGRDELAEVAARQMRELPYYNTFFKTTHVPAIALAQK
LAELAPGDLNHVFFAGGGSEANDTNIRMVRTYWQNKGQPEKNVIISRKNAYHGSTVGSSSLGGMAGMHAQGGIPIPDIHHINQPNWWAEGRPGDTGMTPEEFGLA
RARELEEAILELGEDRVAAFIAEPVQGAGGVIVAPDTYWPEIQRICDKYEILLIADEVICGFGRTGNWFGSQTMGIRPHIMTIAKGLSSGYAPIGGSIVCDEVAEVIIGGKD
EFNHGYTYSGHPVAAAVALENLRILEEENIIDHVRNVAAPYLKEKWEALTDHPLVGEAKIVGMMGSIALTPDKATRAKFASEPGTIGYICRERCFANNLIMRHVGDRMII
SPPLVITPAEIDEMFVRIRKSLDEAHAEILKQGLMKSAG*

>N50
MSMSTITNHMPTAELQALDAAHHMHPFSTNNELAEEGVRVITRAKGVWLTDSEGEEILDAMAGLWCVNIGYGRDELAEVAARQMRELPYYNTFFKTTHVPAIALAQK
LAELAPGDLNHVFFAGGGSEANDTNIRMVRTYWAEKGKPEKNVIISRKNAYHGSTVGSASLGGMAGMHAQGGIPIPDIHHINQPNWWAEGRPGDTGMTPEEFGLAR
ARELEEAILELGEDRVAAFIAEPVQGAGGVIVAPDTYWPEIQRICDKYEILLIADEVICGFGRTGNWFGSQTMGIRPHIMTIAKGLSSGYAPIGGSIVCDEVAEVIIGGKCE
FNHGYTYSGHPVAAAVALENLRILEEENIIDHVRNVAAPYLKEKWEALTDHPLVGEAKIVGMMGSIALTPDKATRAKFASEPGTIGYICRERCFANNLVMRHVGDRMII
SPPLVITPEEIDEMFVRIRKSLDEAHAEILRQGLMKAAG*

>N49
MSMSTITNHMPTAELQALDAAHHMHPFTTNNELAEKGARVITRAKGVWLTDSEGEEILDAMAGLWCVNIGYGRDELAEVAARQMRELPYYNTFFQTTHVPAIALAAK
LAELAPGDLNHVFFAGGGSEANDTNIRMVRTYWAEKGKPEKNIIISRKNAYHGSTVGSASLGGMAGMHAQGGLPIPDIHHIDQPNWWAEGRPGDTGMTPEEFGLAR
ARELEQAILELGEDRVAAFIAEPVQGAGGVIVAPDTYWPEIQRICDKYEILLIADEVICGFGRTGNWFGSQTMGIRPHIMTIAKGLSSGYAPIGGSIVCDEVAEVIIGGKC
EFNHGYTYSGHPVAAAVALENLRILEEENIIDHVRNVAAPYLKEKWEALTDHPLVGEAKIVGMMGSIALTPDKATRAAFASEPGTVGYICRERCFANNLVMRHVGDRM
IISPPLVITPEEIDTLIERARKSLDEAHAELKRQGLMKAAG*

>N48
MSMNTITNHMPTAELQALDAAHHMHPFTTNNELAEKGARVITRAKGVWLTDSEGNEILDAMAGLWCVNIGYGRDELAEVAARQMRELPYYNTFFQTTHVPAIALAAK
LAELAPGDLNHVFFAGSGSEANDTNIRMVRTYWAEKGKPEKNIIISRKNAYHGSTVGSASLGGMAGMHAQGGLPIPDIHHIDQPNWWAEGRPGDTGMTPEEFGLAR
ARELEQAILELGEDRVAAFIAEPVQGAGGVIVPPDTYWPEIQRICDKYEILLIADEVICGFGRTGNWFGSQTMGIRPHIMTIAKGLSSGYAPIGGSIVCDEVAEVIIGGAC
EFNHGYTYSGHPVAAAVALENLRILEEENIIDHVRDVAAPYLKEKWEALADHPLVGEAKIVGMMGSIALTPDKATRAAFASDAGTVGYICRERCFANNLVMRHVGDRM
IISPPLVITPEEIDTLIERARKSLDEAYAELKAEGLMKAAG*
